# Gut Colonization by ESBL-Producing Escherichia coli in Dogs Is Associated with a Distinct Microbiome and Resistome Composition

**DOI:** 10.1128/spectrum.00063-23

**Published:** 2023-07-05

**Authors:** Paul B. Stege, Joost Hordijk, Arnar K. S. Sandholt, Aldert L. Zomer, Marco C. Viveen, Malbert R. C. Rogers, Moniek Salomons, Jaap A. Wagenaar, Lapo Mughini-Gras, Rob J. L. Willems, Fernanda L. Paganelli

**Affiliations:** a Department of Medical Microbiology, UMC Utrecht, Utrecht University, Utrecht, The Netherlands; b Centre for Infectious Disease Control Netherlands, National Institute for Public Health and the Environment (RIVM), Bilthoven, the Netherlands; c Department of Infectious Diseases and Immunology, Faculty of Veterinary Medicine, Utrecht University, Utrecht, The Netherlands; d Utrecht University, Institute for Risk Assessment Sciences, Utrecht, The Netherlands; e WHO Collaborating Centre for Reference and Research on Campylobacter and Antimicrobial Resistance from an One Health Perspective/OIE Reference Laboratory for Campylobacteriosis, Utrecht, The Netherlands; Michigan State University

**Keywords:** ESBL, microbiome, resistome

## Abstract

The gut microbiome of humans and animals acts as a reservoir of extended-spectrum beta-lactamase-producing Escherichia coli (ESBL-EC). Dogs are known for having a high prevalence of ESBL-EC in their gut microbiota, although their ESBL-EC carrier status often shifts over time. We hypothesized that the gut microbiome composition of dogs is implicated in ESBL-EC colonization status. Therefore, we assessed whether ESBL-EC carriage in dogs is associated with changes in the gut microbiome and resistome. Fecal samples were collected longitudinally from 57 companion dogs in the Netherlands every 2 weeks for a total of 6 weeks (*n* = 4 samples/dog). Carriage of ESBL-EC was determined through selective culturing and PCR and in line with previous studies, we observed a high prevalence of ESBL-EC carriage in dogs. Using 16s rRNA gene profiling we found significant associations between detected ESBL-EC carriage and an increased abundance of *Clostridium sensu stricto 1*, *Enterococcus*, *Lactococcus*, and the shared genera of Escherichia*-Shigella* in the dog microbiome. A resistome capture sequencing approach (ResCap) furthermore, revealed associations between detected ESBL-EC carriage and the increased abundance of the antimicrobial resistance genes: *cmlA*, *dfrA*, *dhfR*, *floR*, and *sul3*. In summary, our study showed that ESBL-EC carriage is associated with a distinct microbiome and resistome composition.

**IMPORTANCE** The gut microbiome of humans and animals is an important source of multidrug resistant pathogens, including beta-lactamase-producing Escherichia coli (ESBL-EC). In this study, we assessed if the carriage of ESBL-EC in dogs was associated with changes in gut composition of bacteria and antimicrobial resistant genes (ARGs). Therefore, stool samples from 57 dogs were collected every 2 weeks for a total of 6 weeks. Sixty eight percent of the dogs carried ESBL-EC during at least one of the time points analyzed. By investigating the gut microbiome and resistome composition, we observed specific changes at time points when dogs were colonized with ESBL-EC compared to time points whenESBL-EC were not detected. In conclusion, our study highlights the importance to study the microbial diversity in companion animals, as gut colonization of particular antimicrobial resistant bacteria might be an indication of a changed microbial composition that is associated with the selection of particular ARGs.

## INTRODUCTION

In the last decade, the global emergence of extended-spectrum beta-lactamase-producing Escherichia coli (ESBL-EC) is compromising the efficacy of antimicrobial therapy and increasing the chance of therapy failure (1). The main reservoir of ESBL-EC is the gut microbiome of healthy humans and animals. Companion animals, often considered family members in households, may be an important source of multidrug-resistant organisms, including ESBL-EC (2–4). The most frequently observed beta-lactamase genes in ESBL-EC in the human gut microbiome include: *bla*_CTX-M-15_, followed by *bla*_CTX-M-1_, *bla*_CTX-M-14_, *bla*_CTX-M-27,_ and *bla*_CTX-M-3_ (5, 6). This partially overlaps with the occurrence of beta-lactamase genes in the dog gut microbiome, which include *bla*_CTX-M-1_, followed by *bla*_CTX-M-14_, *bla*_CTX-M-15_, *bla*_SHV-12_, *bla*_CMY-2_ (3, 4). Additionally, van den Bunt et al. detected *bla*_CTX-M-32_ and *bla*_TEM-52_ antimicrobial resistance genes (ARGs) in dogs (7). Longitudinal analysis of ESBL-EC carriage in dogs revealed persistent carriage in 57% of the dogs for 1 month and in 43% of the dogs for 6 months (7). In another study, Baede et al., observed persistent carriage of ESBL-EC in 24% of the dogs studied (3). However, both studies also reported temporal shifts in ESBL-EC carrier status, with the vast majority of dogs shifting over time between positive and negative ESBL-EC carrier statuses, some even showing three or more shifts in a 6-week period (3, 7). These shifts probably reflect significant differences in colonization levels of ESBL-EC in dogs, potentially leading to differences in sensitivity levels of detecting ESBL-EC colonization, or uptake or loss of ESBL-EC strains caused by factors not well understood (3).

The composition of the gut microbiome of dogs is potentially one of the factors involved in ESBL-EC colonization. The intestinal tract of humans and animals is densely colonized by thousands of different species of bacteria that together with fungi, viruses, and phages, represent the gut microbiome (8). This complex ecosystem does not only play a role in host nutrient acquisition but is also involved in health and disease status of the host (9, 10). As a result of the continuous competition for both nutrients and space, commensal gut microbes provide protection against pathogenic bacteria by preventing colonization and subsequent infections, termed colonization resistance (11, 12). This competition, furthermore, drives the gut microbiome toward an equilibrium, despite the daily exposure to new microbes from numerous environmental sources (13–15). The gut microbiome thus represents an important reservoir of ARGs, called the resistome. ARGs that are frequently found as part of the dog intestinal resistome are genes encoding tetracycline resistance (*tet*[W], *tet*[O], *tet*[Q], *tet*[M]), macrolide resistance (*mefA*, *mel*) aminoglycoside resistance (*aph[3″]-Ib*, *ap*[6]*-Id*), lincomycin resistance (*lnuC*), and beta-lactamase resistance (namely, *bla*_OXA-85_) (16).

To unravel whether ESBL-EC carriage in dogs is associated with microbiome and resistome changes, we studied the dog gut microbiome and resistome composition longitudinally in relation to the ESBL-EC status (positive or negative) among 57 dogs, over the course of 6 weeks. We confirmed by culturing, matrix-assisted laser desorption/ionization-time of flight (MALDITOF) and PCR screening that in this study in a large proportion of these dogs (68%) ESBL-EC carriage could be detected. 16S rRNA gene sequencing revealed that detected ESBL-EC carriage is associated with an increased abundance of *Clostridium sensu stricto 1*, *Enterococcus*, *Lactococcus*, and the shared genera of Escherichia*-Shigella*. Target resistome analysis revealed that dogs in which colonization with ESBL-EC was detected also have a higher abundance of *cmlA*, *dfrA*, *dhfR*, *floR*, and *sul3* ARGs. These findings highlight that detected ESBL-EC gut carriage is associated with a distinct gut microbiome and resistome composition.

## RESULTS

### Detected ESBL-EC carriage is associated with specific changes in the gut microbiome.

**FIG 1:**
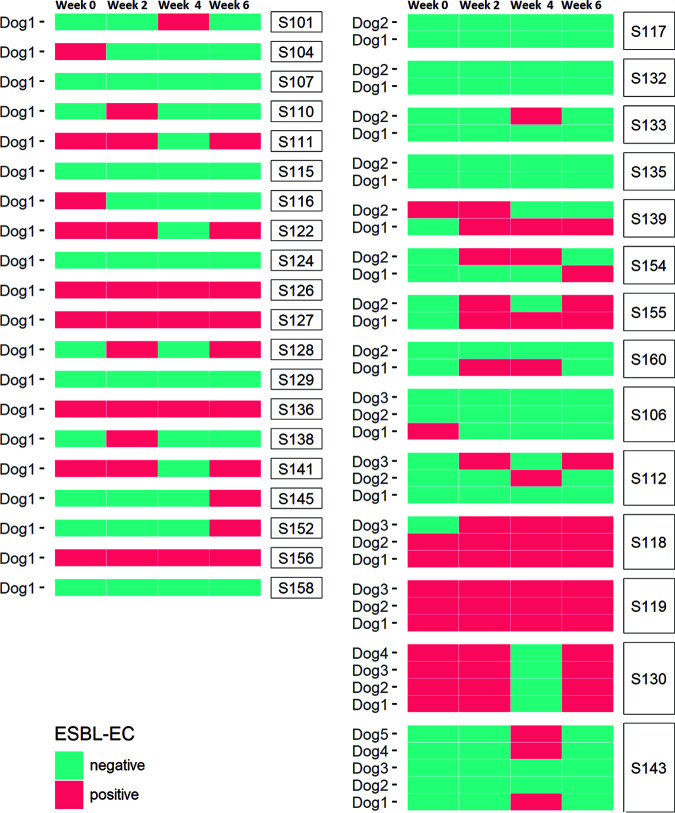
Detection of ESBL-producing Escherichia coli in dogs. Rows represent individual dogs and samples from these dogs are grouped by household, while columns indicate time points with 2-week intervals. S-numbers indicate the households. ESBL-EC detection is indicated in blue when ESBL-EC were detected at a time point, or red when ESBL-EC were not detected.

To investigate the association between carriership of extended-spectrum beta-lactamase-producing Escherichia coli (ESBL-EC) and gut microbiome composition, fecal samples were collected from 57 companion dogs in the Netherlands every 2 weeks for a total of 6 weeks. From these 57 dogs, 37 were part of households with two or more dogs. In a relatively high number of dogs (39, 68%) ESBL-EC were detected at least once over the course of 6 weeks. In nine dogs ESBL-EC (16%) were detected at all time points, while in 30 dogs (52%) ESBL-EC were detected intermittently. In the remaining dogs (18 dogs, 32%) no ESBL-EC were detected over the course of 6 weeks (Fig. 1).

**FIG 2:**
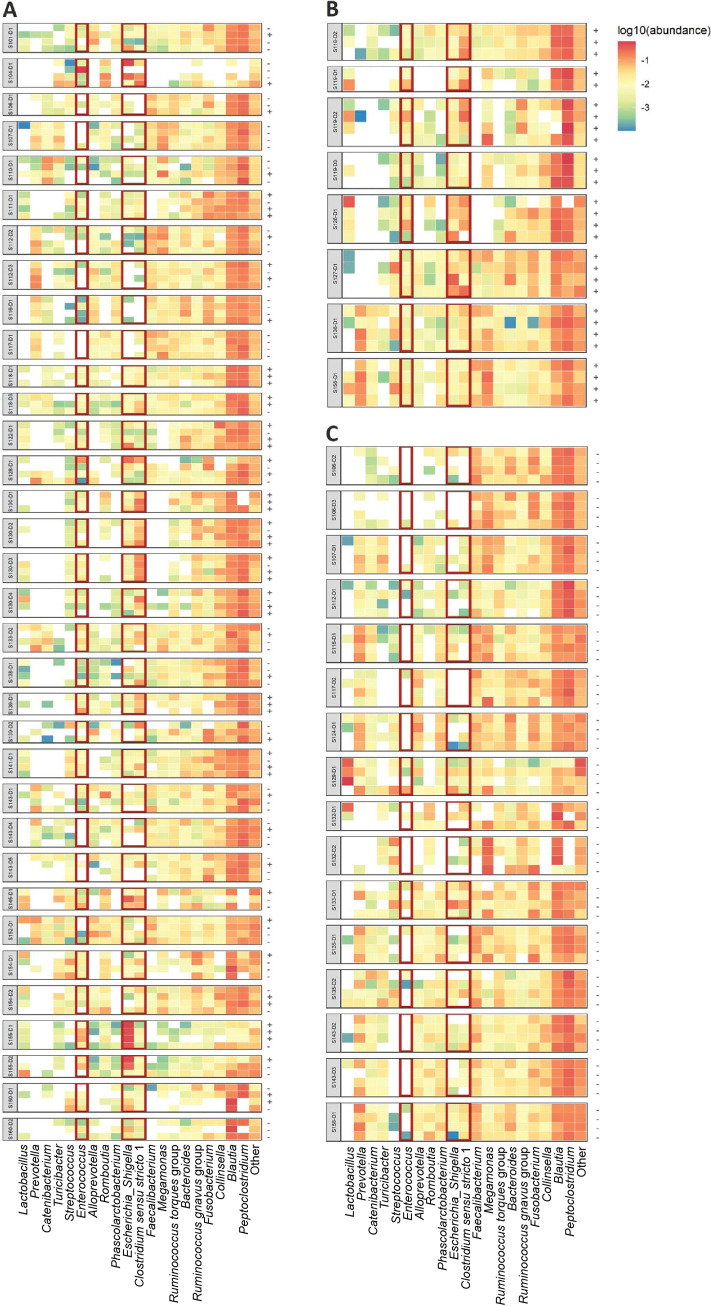
Detected ESBL-EC carriage and the gut microbiome composition. Relative abundance of the 20 most abundant bacterial genera per dog. Each row represents a time point and ESBL-EC detection is marked by “+” when ESBL-EC were detected or “-” when this was not the case. Rows are grouped per dog. (A) Dogs in which ESBL-EC were detected intermittently. (B) Dogs in which ESBL-EC were detected during all time points. (C) Dogs in which ESBL-EC were not detected during at any time point. S-numbers indicate the households and d-numbers the dogs. Red boxes indicate bacterial genera that show large differences in relative abundance between dogs in which ESBL-EC were detected and dogs in which ESBL-EC were not detected during all time points.

To determine gut microbiome composition, 16S rRNA sequencing was performed. The top 20 most abundant bacterial genera in dogs included *Peptoclostridium*, *Blautia*, *Prevotella*, *Faecalibacterium*, and *Bacteroides* (Fig. 2). While individual dogs showed shifts in microbial composition between different time points, the largest systemic difference in gut microbiome composition was observed between dogs in which ESBL-EC carriage was detected at all time points and dogs in which ESBL-EC were not detected (Fig. 2). More specifically, generalized linear mixed-effects models (GLME) revealed that the relative abundance of Escherichia-*Shigella*, *Enterococcus*, and *Clostridium sensu stricto 1* was higher in dogs in which ESBL-EC were detected at all time points compared to dogs where ESBL-EC carriage was not detected during all time points (Fig. 2b and c). These systemic patterns in relative abundance between time points were not as apparent when ESBL-EC carriage was intermittently detected (Fig. 2a).

**FIG 3:**
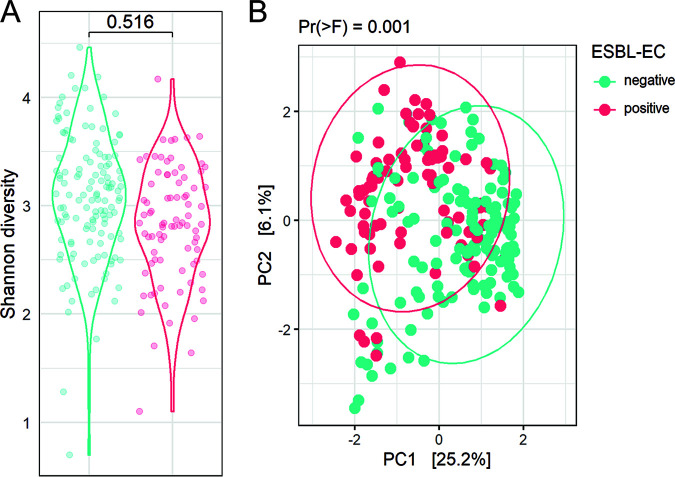
ESBL-EC carriage and the gut microbiome diversity. (A) Alpha diversity expressed by Shannon index and grouped based on dog ESBL-EC carriage. Differences in alpha diversity between groups, expressed by Shannon index, were tested using linear mixed-effects models. (B) Aitchison distance PCA based on bacterial genera with data points and ellipses colored according to dog ESBL-EC carriage, tested with PERMANOVA. ESBL-EC detection is indicated in blue when ESBL-EC were detected at a time point, or red when ESBL-EC were not detected.

Differences in alpha diversity between ESBL-EC groups were analyzed using GLME and the Shannon index. Alpha diversity was not significantly different between dogs in which ESBL-EC were detected in the gut microbiome, or in dogs where this was not the case, indicating that the total species diversity was similar regardless ESBL-EC detection status (Fig. 3a). Principal-component analysis (PCA) based on Aitchison distance was applied to disclose differences in the structure of gut microbiome across all individual dog samples (Fig. 3b). The first two principal components explain a large proportion of the observed variance in gut microbiome composition (31%), suggesting grouping of dogs based on ESBL-EC detection status. Differences were found statistically significant based on permutational multivariate analysis of variance (PERMANOVA) (*P*= 0.001), but not when testing for homogeneity of multivariate dispersions (*P* = 0.053).

**FIG 4:**
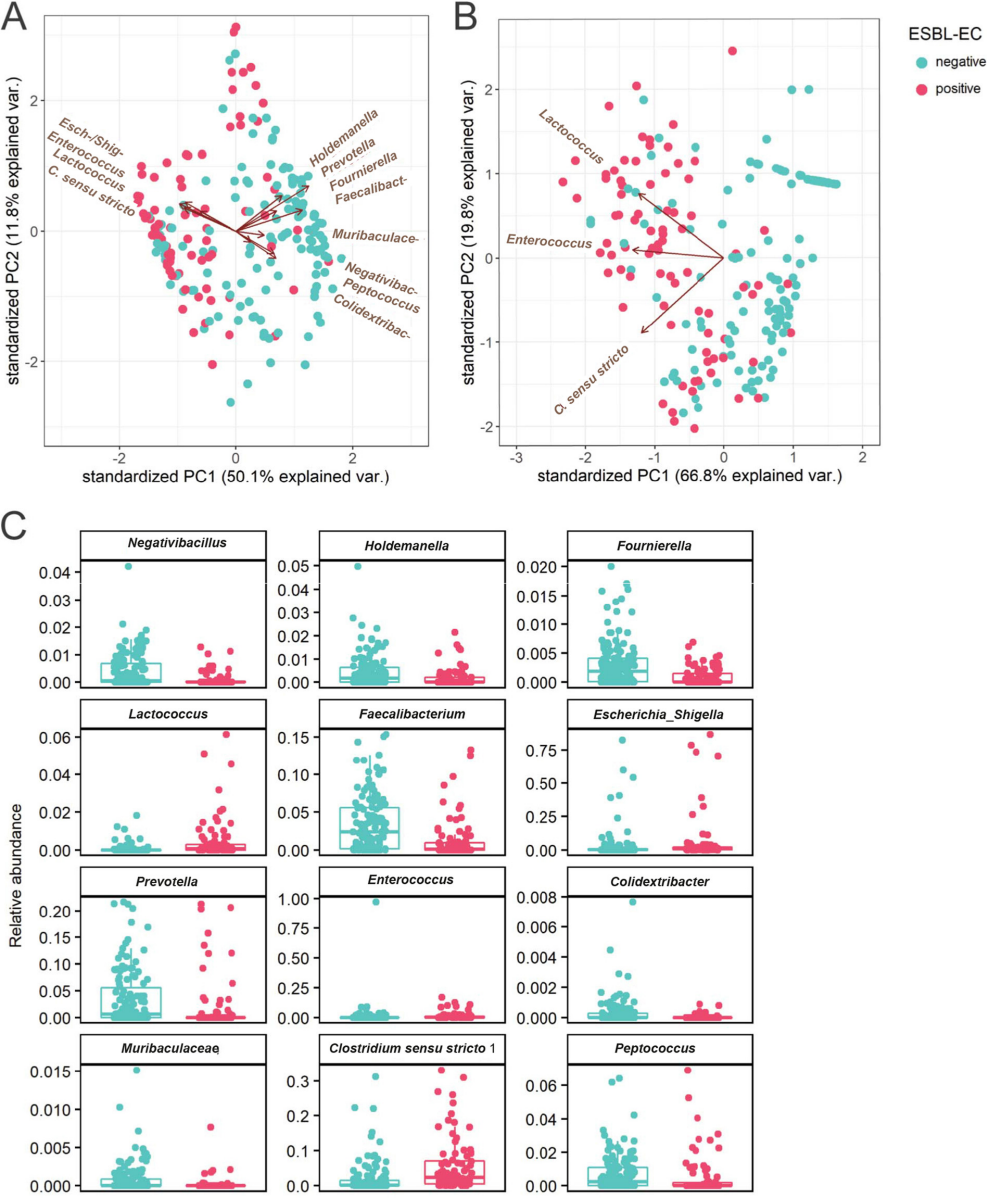
Association of detected ESBL-EC carriage and individual bacterial genera. PCA composed of only the bacterial genera that are significantly associated with detected ESBL-EC carriage or the group where ESBL-EC carriage was not detected, as determined by (A) univariate longitudinal analysis and (B) multivariate longitudinal analysis. (C) Individual abundance of bacterial genera that are significantly associated with detected ESBL-EC carriage. Abundance was plotted on the relative abundance scale from 0.00 to 1.00. ESBL-EC detection is indicated in blue when ESBL-EC were detected at a time point, or red when ESBL-EC were not detected.

To confirm the systemic differences observed in Fig. 2, univariate generalized linear mixed-effects models (GLME) were used to test for associations between the bacterial genera abundance and detected ESBL-EC carriage in dogs Table S1. Univariately, detected ESBL-EC carriage was associated with increased abundance of *Clostridium sensu stricto 1*, *Enterococcus*, *Lactococcus*, and the shared genera of Escherichia*-Shigella* (Fig. 4a, c, Fig. S1). In contrast, detected ESBL-EC carriage was associated with decreased abundance of *Colidextribacter*, *Faecalibacterium*, *Fournierella*, *Holdemanella*, *Muribaculaceae*, *Negativibacillus*, *Peptococcus*, and *Prevotella* (Fig. 4a to c). Multivariate analysis confirmed that the increased abundance of *Clostridium sensu stricto 1*, *Enterococcus* and *Lactococcus*, are together associated with detected ESBL-EC carriage (Fig. 4b and c, Table S1).

### Detected ESBL-EC carriage is associated with the increased abundance of specific antimicrobial resistance genes.

Since the gut microbiome is an important reservoir of antimicrobial resistance genes (ARGs), differences in the observed microbiome composition that were found to be associated with detected ESBL-EC carriage, may also result in changes in the resistome composition. To investigate this, 40 dog fecal samples from 10 households were selected for resistome profiling using resistome capture sequencing approach (ResCap), based on the criteria described in the Materials and Methods (Fig. S2). Single dog households were selected to avoid possible clustering of resistome features among dogs living in the same households. We first set-out to compare the results of ESBL gene detection using ResCap with the detection of ESBL genes using culture-based method followed by conventional PCR screening. In the 40 samples used for ResCap ESBL genes were detected in 19 samples (48%) by PCR (Table S2). In contrast, ResCap detected the ESBL gene *bla*_CTX-M_ in 7/40 (18%) samples. In addition, 1 sample contained *bla*_SHV_, a potential ESBL gene (Table S2). Of note, the applied MEGARes database used in ResCap analysis does not distinguish between different CTX-M groups or SHV variants. In the 14 samples where ResCap did not detect an ESBL gene, but the selective culturing and PCR confirmation did, *bla*_CTX-M-1_ was detected in 7 samples, *bla*_CTX-M-14/18_ in 4 samples, *bla*_CTX-M-15_ in 7 samples, and *bla*_CTX-M-32_ in 2 samples (Table S2). In contrast, ResCap detected *bla*_CTX-M_ ARGs in two samples that were not detected using the culture-based method followed by PCR screening (Table S2).

**FIG 5:**
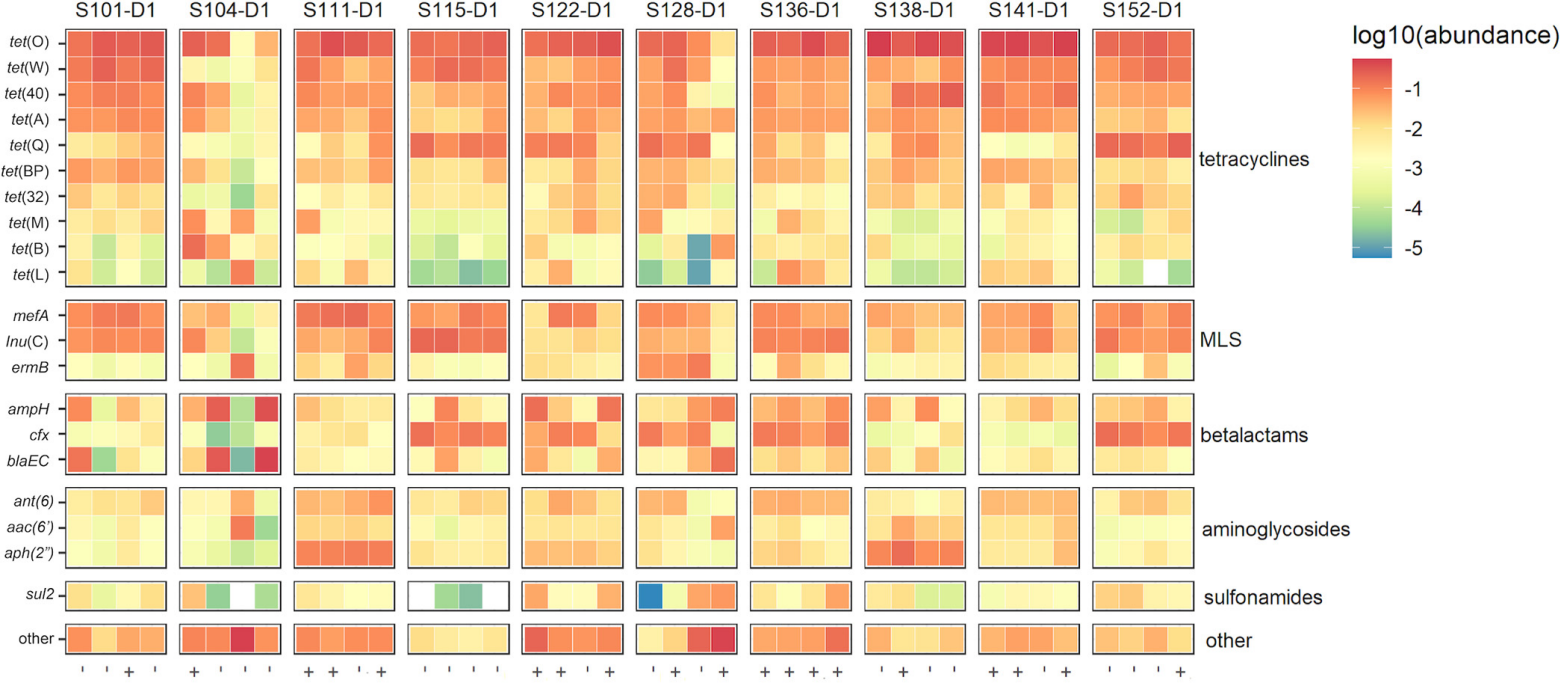
Relative abundance of the gut resistome. Relative abundance of the 20 most abundant ARGs per dog. Each column represents a time point and detected ESBL-EC carriage is marked by “+” or “-” when ESBL-EC carriage was not detected. Columns are grouped per dog and S-numbers indicate the households and d-numbers the dogs.

In total, 133 unique acquired ARGs were identified by ResCap in 40 samples (Table S3). The 20 most abundant ARGs included 10 different tetracycline resistance genes, the macrolides/lincosamides/streptrogramin (MLS) resistance genes *mefA* and *ermB*, lincomycin resistance gene *lnuC*, beta-lactamase resistance genes *ampH*, *cfx*, and *bla*_EC_, aminoglycoside resistance genes *ant* (6), *aph(2′')*, and *aac(6′)*, and sulfonamide resistance gene *sul2* (Fig. 5). No systemic differences were observed in the prevalence of these top 20 most abundant ARGs in dog samples and detected ESBL-EC carriage.

**FIG 6:**
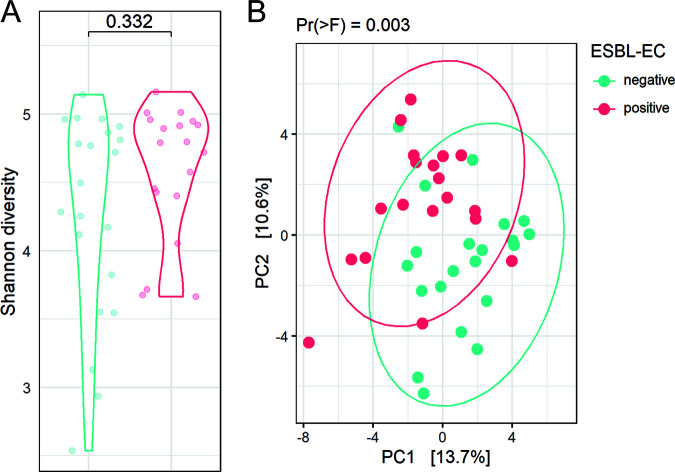
Association of dog gut resistome composition and detected ESBL-EC carriage. Alpha diversity expressed by Shannon index and grouped based on detected ESBL-EC carriage. Differences in alpha diversity between groups, expressed by Shannon index, were tested using LME models. B) Aitchison distance PCA based on bacterial species with data points and ellipses colored according to the detected ESBL-EC carriage status in the dogs, tested with PERMANOVA. ESBL-EC detection is indicated in blue when ESBL-EC were detected at a time point, or red when ESBL-EC were not detected.

The gut resistome alpha-diversity was analyzed using generalized linear mixed-effects models (GLME) and the Shannon index. No significant differences were detected between dog samples in which ESBL-EC carriage was detected and in samples where this was not the case (Fig. 6a, *P* = 0.332). Differences in the beta-diversity of the gut resistome across all individual dog samples were also statistically significant based on PERMANOVA (*P* = 0.003), but not when testing for homogeneity of multivariate dispersions (Fig. 6b, *P* = 0.282).

**FIG 7:**
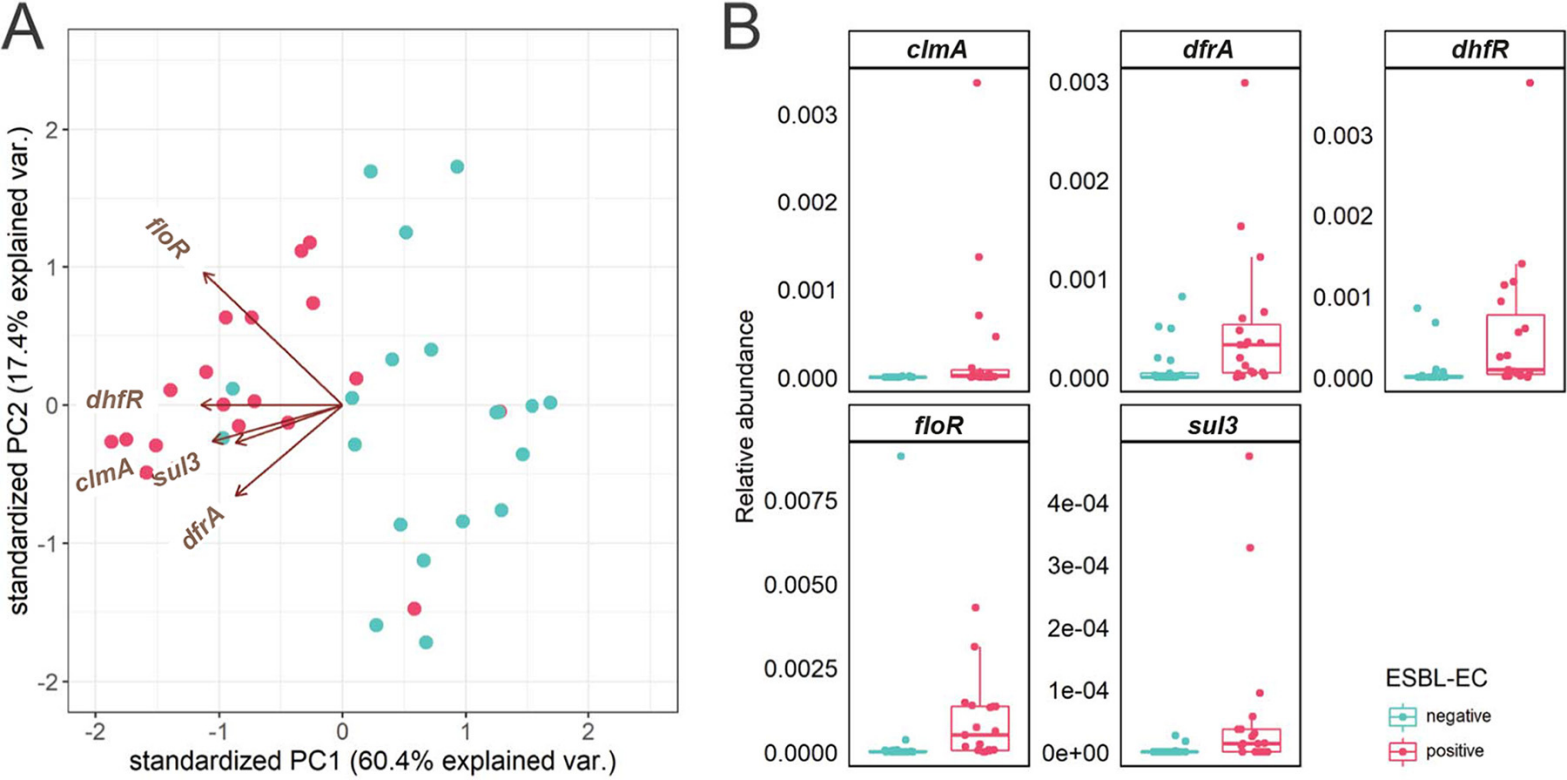
Association of detected ESBL-EC carriage and the resistome. PCA composed of only the ARGs that are significantly associated with detected ESBL-EC carriage, as determined by (A) univariate longitudinal analysis. (B) Individual abundance of ARGs that are significantly associated with detected ESBL-EC carriage. Abundance was plotted on the relative abundance scale from 0.00 to 1.00. ESBL-EC detection is indicated in blue when ESBL-EC were detected at a time point, or red when ESBL-EC were not detected.

Univariate GLME analysis showed significant associations between detected ESBL-EC carriage and increased abundance of the *cmlA*, *dfrA*, *dhfR*, *floR*, and *sul3* genes (Fig. 7a, Table S4). Multivariate analysis confirmed that the increased abundance of *dhfR* was associated with detected ESBL-EC carriage (Table S4). Although *dhfR* was present in low abundance in the resistome, the difference in gene abundance of *cmlA*, *dfrA*, *dhfR*, *floR, and sul3* was mainly due the presence of these genes in dogs in which ESBL-EC carriage was detected, compared to an absence of these genes in dogs were ESBL-EC carriage was not detected (Fig. 7b, Fig. S3).

### Long-read metagenomics of two dog samples.

To obtain more detailed information on genetic context of ARGs, fecal samples of two dogs belonging to households S111 and S128 were subjected to long-read metagenomic sequencing. Long-read metagenomics of fecal DNA yielded 13.5 million reads (sample median of 1.7 million reads) with an *N*_50_ of 2839 bp (total throughput: 17.8 Gbp).

Metagenomic analysis revealed in the dog of household S128 colocalization of some of identified ARGs by ResCap. The florfenicol resistance gene *floR* colocalized with the trimethoprim resistance gene *dfrA36*, the sulfonamide resistance gene *sul2* and the class 1 integron specific recombinase *intI1* on a single 13902 bp read (Fig. S4). In the dog of household S111, the trimethoprim resistance gene *dfrA1* appeared to be colocalized with the streptothricin resistance gene *sat2_gen*, the streptomycin resistance gene *aadA1*, the beta-lactam resistance gene *bla*_TEM-10_, and the sulfonamide resistance gene *sul2* on a single 11439 bp read (Fig. S4).

## DISCUSSION

In this longitudinal study, we assessed carriage of extended-spectrum beta-lactamase-producing Escherichia coli (ESBL-EC) in dogs and determined whether detected ESBL-EC carriage is associated with distinct changes in gut microbiome and resistome composition. Detection of ESBL-EC carriage was highly prevalent in dogs, with 68% of the dogs found positive for ESBL-EC at least once over the course of 6 weeks. This high prevalence of ESBL-EC in dogs is in line with previous findings where 84% of the dogs carried ESBL-producing Enterobacterales over the course of 6 months (3). Furthermore, we observed persistent detection of carriage of ESBL-EC during 6 weeks of time in 16% of the dogs, which is slightly lower than described in previous longitudinal studies where 24% and 43% of dogs were found positive for ESBL-producing Enterobacterales (3, 7). In the current study, in a large percentage of dogs (44%) ESBL-EC carriage was detected intermittently during the study. This is in concordance with previous findings where 61% of the dogs switched between ESBL-EC carriage and, to a lesser extent, with others where 18% of the dogs switched between ESBL-EC carriage (3, 7).

Factors that explain intermittent detection of ESBL-EC carriage by dogs, i.e., fluctuation in the relative abundance of ESBL-EC over time, are not well understood. We hypothesized that the composition of the gut microbiome might be one of the factors explaining this. We set out to study the gut microbiome and resistome composition using feces samples from dogs in which ESBL-EC were detected or not using both 16S rRNA sequencing and ResCap, respectively. Within the selection of Dutch companion dogs, we were able to detect 244 different bacterial genera and 133 unique acquired antimicrobial resistance genes (ARGs) in the dog gut microbiome. The most abundant bacterial genera in dogs included *Peptoclostridium*, *Blautia*, *Prevotella*, *Faecalibacterium*, and *Bacteroides*, which was in line with gut microbiome diversity observed in dogs in previous studies (13–15). Comparing the gut microbiome of dogs in which ESBL-EC were detected to dogs where this was not the case at the time of sampling, revealed associations between detected ESBL-EC carriage and an increased abundance of the bacterial genera *Clostridium sensu stricto 1*, *Enterococcus*, *Lactococcus*, and the shared genera of Escherichia*-Shigella.* Multivariate analysis confirmed the observed associations of *Clostridium sensu stricto 1*, *Enterococcus*, and *Lactococcus* with detected ESBL-EC carriage.

Detected ESBL-EC carriage was associated with decreased abundance of *Colidextribacter*, *Faecalibacterium*, *Fournierella*, *Holdemanella*, *Muribaculaceae*, *Negativibacillus*, *Peptococcus*, and *Prevotella*. Some species of *Faecalibacterium*, *Holdemanella*, and *Prevotella* are considered beneficial to the host (17–20). Similarly, *Muribaculaceae*, *Negativibacillus*, and *Peptococcus* are associated with short-chain fatty acid (SCFA)-production (21–23). Currently little is known about *Colidextribacter* and *Fournierella* (24). The only cultured species of *Fournierella* is *F. massiliensis*, which may have a beneficial role in the gut of its host through butyrate production (25).

These results show that detection of ESBL-EC carriage in dogs is associated with a specific microbiome signature.

In humans, conflicting results were found when studying the role of the gut microbiome in ESBL-EC carriage. When studying 98 individuals from the Dutch population, Ducarmon et al. did not detect differences in microbiome diversity or in relative abundance of bacterial species between ESBL-positive and ESBL-negative groups in a matched case-control study (26). This is in contrast to the findings of Le Bastard et al., when studying the residents of French nursing homes. In that study, carriage of extended beta-lactamase producing Enterobacteriaceae was associated with an increase of Bifidobacterium animalis and three *Prevotella* species and with a decrease of Clostridium hylemonae, Collinsella tanakaei, Johnsonella ignava, and Bifidobacterium adolescentis (27). The lack of consensus between studies could be related to differences between the study groups, including geographic location, age and antibiotic use of participants. An important difference between these previously published studies in humans and the design of our study is that the gut microbiome composition of the human participants was not studied over time and intraindividual variability of the microbiome could therefore hinder the detection of ESBL-EC associated compositional changes in the gut microbiome.

To investigate whether a distinct microbiome signature also affects the composition of ARGs we applied ResCap to investigate the gut resistome in dogs. We first compared the sensitivity of ResCap to detect ESBL genes to that of culture-based method, followed by conventional PCR screening. While culturing and PCR was able to detect ESBL genes in 19 out of 40 samples (48%), ResCap was able to detect ESBL genes in only 7 samples (18%). Most probably, the low abundance of these genes explains this discrepancy and is a limiting factor for detecting these genes by ResCap which is line with a recent publication showing also low abundance of ESBL encoding genes in both ESBL-negative and ESBL-positive humans in a Dutch cross-sectional population-wide study (26). On the other hand, using ResCap we were able to detect *bla*_CTX-M_ resistance genes in two samples in which ESBL genes were not detected using the culture-based method followed by PCR screening. Explanations for this discrepancy might be that in these samples the *bla*_CTX-M_ gene is carried by bacteria that were not selected during the culture step, because they are for instance strict anaerobes or because *bla*_CTX-M_ genes were not expressed.

Resistome analyses revealed associations between the abundance of specific ARGs and ESBL-EC carriage detection. These ARGs encode resistance to trimethoprim (*dfrA*, *dhfR*), phenicol (*cmlA*, *floR*), and sulfonamides (*sul3*). Multivariate analysis confirmed the observed associations of *dhfR* with detected ESBL-EC carriage. The combination of trimethoprim and sulfamethoxazole is a widely used clinical and veterinary medicine to treat bacterial infections and coresistance is commonly observed in ESBL-producing *Enterobacterales* (28). Specifically, the genes *sul1*, *sul2*, *sul3*, *dfrA*, and *dhfr* are commonly located on plasmids and are found in close association with class 1 integrons or ISCR mobile genetic elements (29–31). While *floR* is also commonly observed in *Enterobacterales*, it specifically encodes resistance to florfenicol, which is a derivative of chloramphenicol and used almost exclusively for treatment of infections in aquaculture and livestock (32–35). In a similar way, *cmlA* encodes resistance to chloramphenicol which is associated with class 1 integrons and carried on plasmids in *Enterobacterales* (36, 37).

Metagenomic Nanopore sequencing reveals that the gut microbiome of dogS111 contains genes *dfrA1*, *sat2_gen*, *aadA1*, *bla*_TEM-10_, *sul2*, that are located on a single genetic element. For dogS128 ARGs *floR*, *dfrA36*, *sul2*, and the class 1 integron specific recombinase *intI1* are located on a single genetic element, which is in concordance with previous findings, and may result in the increased circulation of ARGs in the environment through coselection (28, 38).

To conclude, our findings suggest that the gut microbiome composition of dogs is implicated in ESBL-EC carriage detection in dogs. It is yet unknown if these changes in the gut microbiome and resistome facilitate ESBL-EC colonization levels or are driven by ESBL-EC colonization levels. Previous findings indicate that diet has a greater impact on the gut microbiome composition than dog breed, age or weight and that consumption of raw meat increases the risk of ESBL-EC carriage, compared to a diet of dry food (3, 39). Raw meat diets were also found to increase abundances of *Lactobacillus* and *Clostridium* in the dog gut microbiome (13, 40).

A limitation of our study is that data on dietary habits for the dogs included in this study were unfortunately not available. Furthermore, other metadata like the breed and age of the dogs were lacking. In addition, data on the taxonomic composition of samples were based on 16S rRNA sequencing, allowing analyses of the distribution and relative abundance of taxa only on genus-level. Alternative metagenomic sequencing techniques like long-read sequencing are able to surpass this level of resolution and allow for species-specific associations. We here applied long-read sequencing as a proof of principle, but the limited number of sequenced samples only allowed providing genetic context of some of the detected antibiotic resistance genes but not a detailed comparison of the taxonomic composition of samples of dogs that carried ESBL-E. coli and dogs that did not at species or subspecies level.

Co-enrichment of antimicrobial-resistant bacteria from different species, such as ESBL-EC, *Clostridium*, *Enterococcus*, and *Lactococcus*, as we found in this study, highlights the importance to study the microbial and resistome composition in companion animals. Gut colonization of antimicrobial resistant bacteria, like E. coli, might be an indication of more profound changes in gut microbiome composition in which multiple antimicrobial resistant bacteria are enriched. This may pose a risk for zoonotic transmission of antimicrobial resistance bacteria and genes from companion animals to humans.

## MATERIALS AND METHODS

### Ethics statement.

Animal sampling was performed in accordance with the guidelines of the Dutch Animals Act (stb-2011-345) and the Animal Welfare Body Utrecht. No additional license was required.

### Household inclusion.

In this study, fecal samples were collected from 57 dogs that were part of 34 households in the general Dutch population (Fig. 1). The included households were part of a database of participants from a prior study on parasites in household dogs (41, 42). The participating households from the original study were recruited again through a request posted on social media and through dog owner support groups. All participating households in the original database who had not given objection to be approached for potential future research projects were addressed to participate in the present study. All communication was by e-mail. All participating households received an invitation letter and additional information sheet explaining the topic and the goals of the project. Four samples were collected longitudinally per participant, using 2-week intervals for a total of 6 weeks. Samples were collected over a 5-month period. No underlying health conditions were reported for the participating dogs at the time of sample collection.

### Sample collection, storage, and DNA extraction.

All participants received a sampling package with instructions on how to collect the samples short before the planned sampling moment. fecal samples were sent by the dog owner by regular mail at room temperature, which is typically delivered within 24 to 48 h. Upon arrival at the lab samples were used for selecting culture ESBL (described in the next section in details), while the remaining fecal material was stored at −80°C. One freeze-thaw cycle was introduced when dividing samples into aliquots of 0.2 mL. Aliquoted samples were thawed a second time for DNA extraction, using a modified protocol of the QIAamp fast DNA stool minikit (Qiagen, Venlo, the Netherlands). In brief, 0.2 g feces were added to 500 μL 0.1 mm zirconium beads (Lab Services) and 1 mL InhibitEx buffer (Qiagen) in a 2 mL Sarstedt tube. Beat beating was performed two times at 3800 rpm for 2 min, using a Mini-beadbeater-24 (Biospec, Rijswijk, the Netherlands) and applying 2-min ice cooling steps in between. Samples were subsequently incubated at 95°C for 7 min, followed by 1 min centrifugation at 16.000 × *g*. The supernatant was removed and stored, while 1 mL InhibitEx buffer was added to the bead-beating tube with left over material. The bead-beating, incubation and centrifugation steps were repeated, and the supernatant removed. Both supernatant fractions were treated with proteinase K and pooled by passing both fraction through a single spin column, according to the fast DNA stool minikit protocol (Qiagen). DNA elution was performed using 100 μL Buffer EB (Qiagen) and DNA LoBind microcentrifuge Eppendorf tubes (VWR International, Amsterdam, the Netherlands). Total DNA was quantified by Picogreen assay (Thermo Fisher Scientific, Eindhoven, the Netherlands).

### ESBL-EC characterization.

Fecal samples were used to inoculate MacConkey agar plate supplemented with 1 mg/L cefotaxime (MacC+) and incubated overnight aerobically at 37°C, in order to select for third generation cephalosporin resistant bacteria. In addition, 0.5 g fecal material was suspended in 4.5 mL LB broth with 1 mg/L cefotaxime (LB+), which was subsequently inoculated on another MacC+ plate after overnight incubation at 37°C. From growth in direct inoculation on the MacC+ plate, five colonies per agar plate were selected for PCR screening for the presence of ESBL genes. When growth was observed in MacC+ after enrichment in LB+, one colony per plate was selected for PCR screening for the presence of ESBL genes (Table S5). When ESBL genes were detected by PCR, the PCR products were sent for Sanger sequencing to determine which ESBL genes were present (BaseClear, Leiden, the Netherlands, Table S6). Bacterial species were further confirmed using Matrix-Assisted Laser Desorption/Ionization Time of Flight Mass-Spectrometry (MALDI-TOF MS). A sample was considered to contain ESBL-producing E. coli, here referred as ESBL-EC, when the presence of ESBL genes and E. coli was confirmed by PCR (table S5) and MALDI-TOF MS, respectively.

### 16S rRNA gene sequencing.

All fecal samples were subjected to 16s rRNA gene sequencing to determine gut microbiome composition after DNA extraction as described above. The 16S rRNA gene hypervariable regions V3 and V4 were amplified (~430 bp) and sequenced with an Illumina MiSeq reagent kit v3 (600-cycles) on a MiSeq system (Illumina, Eindhoven, the Netherlands), as was described previously (43, 44). Sequences were taxonomically assigned using QIIME 2 (version 2018.8), DADA2 (package version 1.16), and the SILVA database 138, applying a cutoff 8000 reads (45–47).

### ResCap sequencing and data processing.

Total DNA extracted from 40 fecal samples were selected for in-depth resistome analysis using the ResCap targeted sequence capture panel consisting of probes targeting 7963 antimicrobial resistance genes (ARGs), and additional probes against the following *mcr* genes (*mcr1.1*, *mcr1.2*, *mcr1.3*, *mcr1.4*, *mcr1.5*, *mcr1.6*, *mcr1.7*, *mcr1.8*, *mcr1.9*, *mcr1.10*, *mcr2.1*, *mcr2.2*, *mcr2.3*, *mcr3.1*, *mcr3.2.1*, *mcr3.2.2*, *mcr3.3.1*, *mcr3.3.2*, *mcr3.4.1*, *mcr3.4.2*, *mcr3.5.1*, *mcr3.5.2*, *mcr3.6*, *mcr4*, *mcr5*, *mcr6*, *mcr7*, and *mcr8*) (Roche ID: OID41815) (48, 49). Samples were selected based on the following criteria: (i) only households that contained a single dog were included; (ii) samples of all four time points were available; and (iii) a total of 1 μg of DNA per sample was available. Based on these criteria 10 households were selected. ResCap was performed according to the supplied protocol. In brief, 600 to 700 ng DNA was used for fragmentation using the KAPA HyperPlus kit v4.17 (Roche, Woerden, The Netherlands) to generate 400 bp fragments. End repair, A-tailing and adapter ligation were performed as described by the SeqCap EZ HyperCap User’s Guide v2.3. Pools of 12 samples were used for hybridization and capture using an extended version of the ResCap probe collection as described in the original publication (48). Sample pools were sequenced on a NovaSeq 6000 using a S1 reagent kit (300 cycles) (Illumina). ResCap-based targeted enriched Illumina reads were trimmed using Trim Galore version 0.6.4 with standard settings (50). KMA version 1.3.4 was used to align sequences to the MEGARes 2.0 database (51, 52) which combines described databases of ARGs, metal and biocide resistance genes. For KMA, paired-end reads were used as input by using *-ipe*, together with the options: *-tmp*, *-1t1*, *-cge*, *-apm p*, *-ef*. The resulting list of detected genes and their abundance was trimmed by applying a cutoff 90% identity (named query Identity) and of 80% coverage (named template coverage). The output value depth was used for subsequent analysis, which represents the amount of aligned base pairs, while correcting for gene length.

### Nanopore sequencing and data processing.

The same DNA extracted from fecal samples used for 16S rRNA profiling and ResCap analysis of the dogs from household S111 and S128 was used for metagenomic Nanopore sequencing. The reason for this selection was the need for representative dogs from the selection of the ResCap. Based on the selection, dogs from households S111 and S128 had intermediate ESBL colonization based on the PCR data that could be confirmed with ResCap (see table S2). In addition, these were the dogs we had enough DNA from to perform nanopore sequencing (1 μg of total DNA). R9.4.1 flow cells were used in combination with the ligation sequencing kit (SQK-LSK109) and native barcode expansion kit (EXP-NBD104, all Oxford Nanopore Technologies, Oxford, UK). Data acquisition and base-calling were performed by MinKNOW version 20.10.6 and demultiplexing by Guppy version 4.0.11.

### Data analysis.

Analysis of sequencing data was performed in R version 4.1.0 and the functions of the packages phyloseq v1.34 and ggplot2 v3.3.3 (53–55). The abundance of the top 10 gut microbiome taxa and ARGs were plotted using aggregate top taxa and plotting functions of the microbiome package v1.12 (56). Shannon index of diversity was calculated using the alpha diversity functions of the microbiome package and plotting functions of the microbiomeutilities package v1.00.09 (57). The package lme4 v1.1-27 was used to apply generalized linear mixed-effects models (GLME). GLME and with logit link function and binomial error distribution were used to test differences in alpha diversity between ESBL-EC groups, accounting for the cluster-longitudinal nature of the data, with the same dogs being sampled over time and some of them belonging to the same households. Aitchison distance PCA was applied using the transform function of the microbiome package and ordinate (RDA) and plot ordination functions of the phyloseq package.

Permutational Multivariate Analysis of Variance (PERMANOVA) and tests on homogeneity of dispersion were employed using the adonis function (999 permutations, seed of 194175) and betadisper function of the vegan package v2.5.7 (58). GLME analysis was also used to test for differentially abundant taxa (genus level) and ARGs associated with ESBL-EC. Normalized 16S rRNA or ResCap data were converted to relative abundance and used for centered log-ratio (CLR) transformation. For the 16S rRNA data, a filtering cutoff 10% prevalence and 0.01% abundance of amplicon sequence variants (ASVs) were applied. The remaining targets were tested for collinearity by means of the variance-inflation factors (VIF) function of the car package v3.0.10, ensuring a VIF < 5 and were assessed with GLME. For GLME analysis of the ResCap data, we applied these same filtering steps, but subsequently selected for acquired ARGs only. To control for multiple-hypothesis testing, the Benjamini–Hochberg method was used. Candidate taxa and ARGs that were associated with ESBL-EC, were selected using univariate analysis (*P* < 0.1). Candidate taxa and ARGs were then entered in a multivariable GLME for ESBL-EC: a backward variable selection procedure was then applied to remove nonsignificant (*P* > 0.05) targets. Nanopore metagenomic data analysis was performed using ABRicate (version 0.9.7) with standard settings to analyze the resistome, using the NCBI database extended with the class 1 integron specific recombinase *intI1* (38, 59).

### Data availability.

The 228 16S rRNA gene sequencing, 40 ResCap sequencing and 8 Nanopore sequencing files have been deposited in the European Nucleotide Archive repository under the study accession PRJEB50027. R scripts to reproduce the analysis reported in this study can be found at https://gitlab.com/PB_Stege/dog_microbiome_resistome.
