# Supplementary material for: Gut Colonization by ESBL-Producing Escherichia coli in Dogs Is Associated with a Distinct Microbiome and Resistome Composition
Source: Microbiol Spectr. 2023 Jul 5;11(4):e00063-23. doi: 10.1128/spectrum.00063-23 (PMC10434115; doi:10.1128/spectrum.00063-23)
Supplement: Supplemental file 1 — Supplemental material. Download spectrum.00063-23-s0001.pdf, PDF file, 0.6 MB [file spectrum.00063-23-s0001.pdf]

Supplementary information for

Gut colonization by ESBL-producing *Escherichia coli* in dogs is associated with a distinct microbiome and resistome composition.

Paul B. Stege[1], Joost Hordijk[2 3], Arnar K. S. Sandholt[4], Aldert L. Zomer[3 5], Marco C. Viveen[1], Malbert R.C. Rogers[1], Moniek Salomons[1], Jaap A. Wagenaar[3 5], Lapo Mughini-Gras[2 4], Rob J. L. Willems[1], Fernanda L. Paganelli[1]

1. *Department of Medical Microbiology, UMC Utrecht, Utrecht University, Heidelberglaan 100, 3584 CX Utrecht, The Netherlands*
2. *Centre for Infectious Disease Control Netherlands, National Institute for Public Health and the Environment (RIVM), Bilthoven, the Netherlands.*
3. *Department of Infectious Diseases and Immunology, Faculty of Veterinary Medicine, Utrecht University, Utrecht, The Netherlands*
4. *Utrecht University, Institute for Risk Assessment Sciences, Utrecht, The Netherlands*
5. *WHO Collaborating Centre for Reference and Research on Campylobacter and Antimicrobial Resistance from an One Health Perspective/OIE Reference Laboratory for Campylobacteriosis, Utrecht, The Netherlands.*

Table s1. Univariate and multivariate longitudinal gut microbiome analysis.

	median abundance (%) ESBL-EC		univariate		multivariate	
**Genus**	**positive**	**negative**	**estimate**	**p-adj**	**estimate**	**p-adj**
Clostridium_sensu_stricto_1	0.16	2.38	0.301	0.013	0.205	0.011
Colidextribacter	0.00	0.00	-0.513	0.054		
Enterococcus	0.00	0.49	0.312	0.002	0.178	0.022
Escherichia_Shigella	0.07	0.81	0.246	0.038		
Faecalibacterium	2.40	0.09	-0.246	0.044		
Fournierella	0.19	0.00	-0.291	0.052		
Holdemanella	0.18	0.00	-0.262	0.054		
Lactococcus	0.00	0.07	0.372	0.002	0.265	0.006
Muribaculaceae	0.00	0.00	-0.378	0.054		
Negativibacillus	0.05	0.00	-0.261	0.052		
Peptococcus	0.23	0.00	-0.216	0.054		
Prevotella	0.65	0.00	-0.218	0.044		

Table s4. Univariate and multivariate longitudinal resistome analysis.

	median abundance (%) ESBL-EC		univariate		multivariate	
**Antibiotic resistance gene**	**positive**	**negative**	**estimate**	**p-adj**	**estimate**	**p-adj**
cmlA	0.00	0.00	0.480	0.082		
dfrA	0.00	0.03	0.359	0.082		
dhfR	0.00	0.01	0.490	0.065	0.490	0.002
floR	0.00	0.05	0.287	0.082		
sul3	0.00	0.00	0.434	0.082		

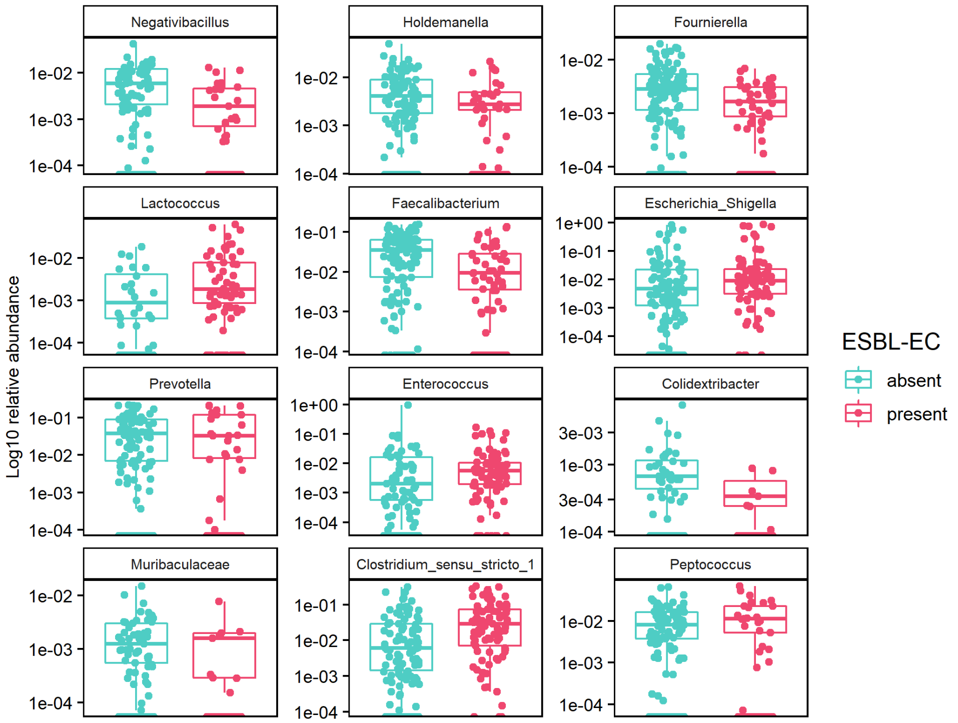

Figure s1. Individual abundance of bacterial genera that are significantly associated with detected ESBL-EC carriage. Abundance was plotted on log10 scale. ESBL-EC detection is indicated in blue when ESBL-EC were detected at a time point, or red when ESBL-EC were not detected.

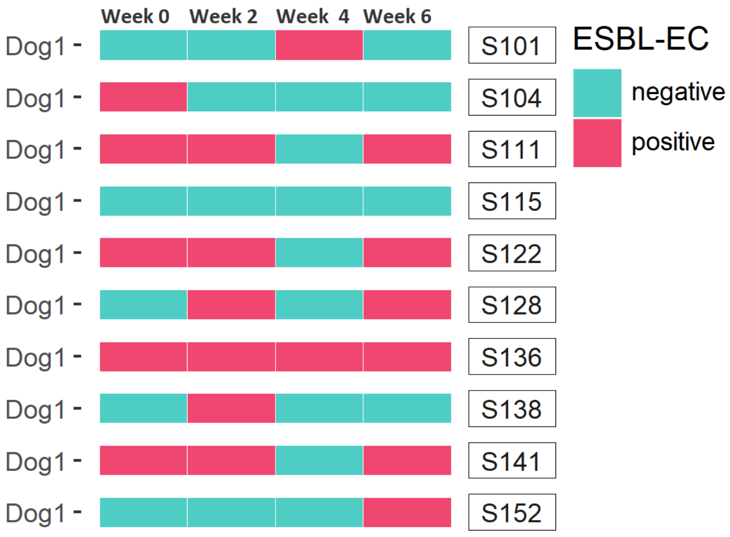

Figure s2. Detected carriage of ESBL-producing *Escherichia coli* in dogs selected for ResCap analysis. Rows represent individual dogs and S-numbers indicate the households, while columns indicate time points with two-week intervals. ESBL-EC detection is indicated in blue when ESBL-EC were detected at a time point, or red when ESBL-EC were not detected.

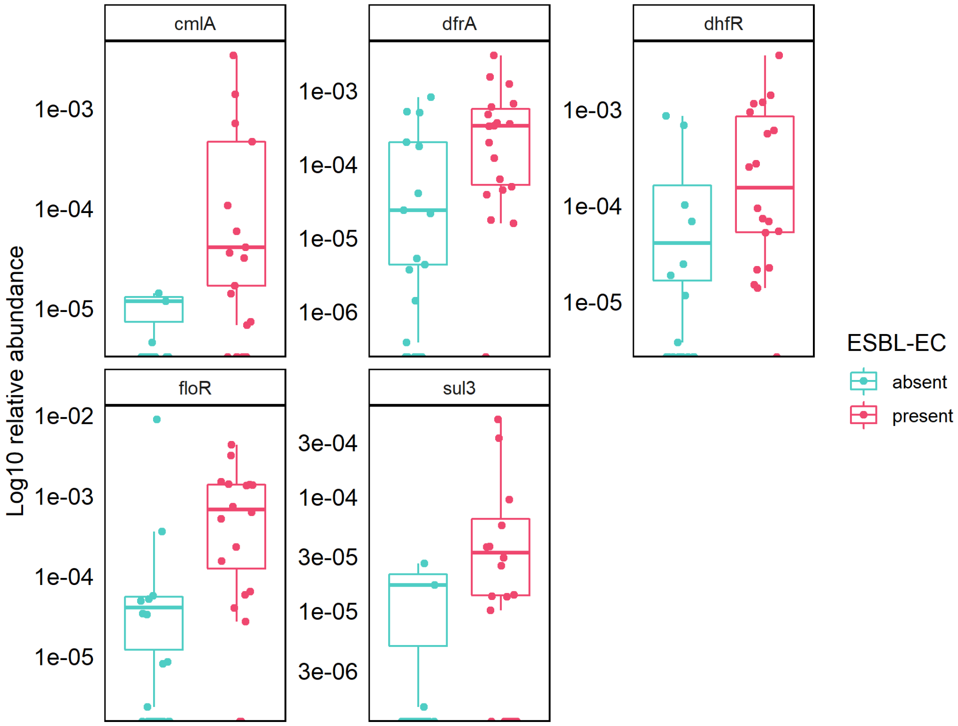

Figure s3. Individual abundance of antimicrobial resistance genes that are significantly associated with detected ESBL-EC carriage. Abundance was plotted on log10 scale. ESBL-EC detection is indicated in blue when ESBL-EC were detected at a time point, or red when ESBL-EC were not detected.

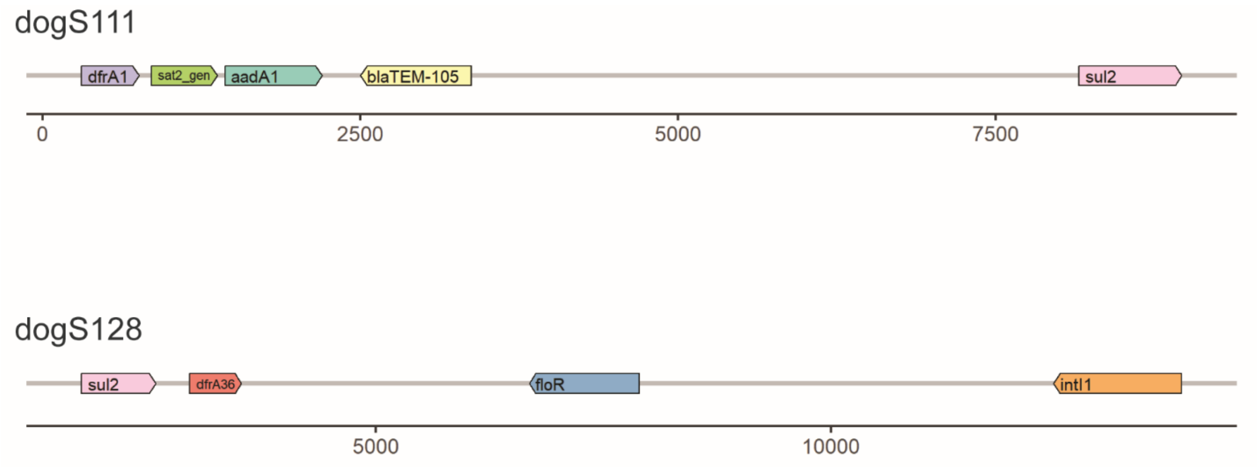

Figure s4. Antibiotic resistance gene context. Nanopore metagenomic sequencing revealed that dogS111 contained ARGs *dfrA1*, *sat2_gen*, *aadA1* and *bla*$_{\text{TEM-10}}$ in close physical distance on a single sequencing read. DogS128 contained resistance genes *floR*, *dfrA36*, *sul2*, and the class 1 integron specific recombinase *intI1*, located on a single sequencing read.
